# Competitive interactions as a mechanism for chemical diversity maintenance in *Nodularia spumigena*

**DOI:** 10.1038/s41598-021-88361-x

**Published:** 2021-04-26

**Authors:** Sandra Lage, Hanna Mazur-Marzec, Elena Gorokhova

**Affiliations:** 1 Department of Environmental Science, Stockholm University, Stockholm, Sweden; 2 Division of Marine Biotechnology, Institute of Oceanography, University of Gdańsk, Gdynia, Poland

**Keywords:** Chemical ecology, Metabolomics, Natural products, Peptides, Target identification, Ecophysiology, Microbial ecology

## Abstract

*Nodularia spumigena* is a bloom-forming diazotrophic cyanobacterium inhabiting brackish waters worldwide. This species produces non-ribosomal peptides (NRPs), including the hepatotoxin nodularin, often referred to as cyanotoxin. Several known classes of NRPs have various biological activities, although their modes of action are poorly understood. In the Baltic *N. spumigena*, there is a high NRP chemodiversity among strains, allowing their grouping in specific chemotypes and subgroups. Therefore, it is relevant to ask whether the NRP production is affected by intraspecific interactions between the co-existing strains. Using a novel approach that combines culture technique and liquid chromatography-tandem mass spectrometry for the NRP analysis, we examined *N. spumigena* strains under mono- and co-culture conditions. The test strains were selected to represent *N. spumigena* belonging to the same or different chemotype subgroups. In this setup, we observed physiological and metabolic responses in the test strains grown without cell contact. The changes in NRP levels to co-culture conditions were conserved within a chemotype subgroup but different between the subgroups. Our results suggest that intraspecific interactions may promote a chemical diversity in *N. spumigena* population, with higher NRP production compared to a single-strain population. Studying allelochemical signalling in this cyanobacterium is crucial for understanding toxicity mechanisms and plankton community interactions in the Baltic Sea and other aquatic systems experiencing regular blooms.

## Introduction

*Nodularia spumigena* is a filamentous diazotrophic cyanobacterium that dominates summer blooms in the Baltic Sea^1^. It is also common in brackish waters worldwide, mainly in Australia and New Zealand^2^, as well as inland saline lakes and ponds on the North and South American and African continents^3–5^. Moreover, due to climate-related and anthropogenic alterations in aquatic environments, cyanobacterial blooms are predicted to increase in frequency and intensity^6^. Blooms of *N. spumigena* are harmful, primarily associated with the production of cyclic pentapeptide nodularin (NOD), posing a risk to animal and human health^7,8^. However, NOD is not the only non-ribosomal peptide (NRP) produced by this cyanobacterium. Similar to other cyanobacteria, *N. spumigena* produces a wide range of structurally diverse NRPs and yet uncharacterized molecules^9–11^. These NRPs are synthesized by multifunctional non-ribosomal peptide synthetases (NRPS) or via combined NRPS and polyketide synthases (PKS) pathways^12–16^. The known NRPs have diverse biological properties, including cytotoxic, antiviral, and allelopathic activities^13,17–19^, and their mechanisms of action are often related to inhibition of vital eukaryotic enzymes^18–21^. However, the specific functions and the ecological roles played by these NRPs in natural communities are poorly understood.

Cyanobacteria produce various NRP classes and congeners that are often strain-specific, resulting in NRP profiles that can be used as fingerprints to distinguish morphologically undistinguishable strains as chemotypes^9,12^. Although the NRP profile of a strain is under genetic regulation, the up- or down-regulation of its components might vary in response to environmental cues^11,22,23^.

In *N. spumigena*, five classes of NRPs have been identified: NODs, spumigins (SPUs), aeruginosins (AERs), pseudaeruginosins (NS), and anabaenopeptins (APs)^24^. Twenty five strains of *N. spumigena* isolated from the Baltic Sea were shown to have a high chemodiversity concerning the produced NRP variants and their structure^9^. Based on this diversity, Mazur-Marzec and co-workers classified these strains into two chemotype groups (CT_A and CT_B). Moreover, in CT_B, different sub-groups were found (B1, B2 and B3); notably, the grouping was independent of the strain geographic origin and isolation time (Supplementary Fig. 1)^9^. Moreover, Mazur-Marzec et al*.* suggested that strains that produce structurally different variants of the same NRP class can co-exist both within a bloom event and outside of the bloom^9^. The intriguing question is whether the distribution, growth, photosynthesis, and NRP production in *N. spumigena* are regulated through intraspecific interactions. One can speculate that NRPs could provide some competitive traits to their producer with concomitant effects on inter- and intraspecific interactions and repercussions on the cyanobacterium fitness. Comparing the fitness and NRP production between the strains that belong to various chemotype subgroups and are grown under monoculture and co-culture conditions can reveal whether the intraspecific interactions are in play. The observed responses to the co-culture conditions could also improve our understanding of the NRP ecological functions and dynamics of cyanobacterial metabolites in the environment.

If NRPs are involved in intraspecific interactions between the strains, and these interactions promote metabolic diversity, then we can put forward the following hypotheses: (H1) Physiological and metabolic differences will be observed between the monoculture and co-cultures of *N. spumigena* strains; moreover, a higher NRP concentrations would be expected in at least one strain of the co-cultures; (H2) In the co-cultures, the response will be more pronounced if the co-cultured strains belong to the same subgroup within a chemotype compared to those belonging to different subgroups; (H3) Strains of the same chemotype subgroup will respond similarly to the co-culture treatments; and (H4) NRP levels are positively related to the physiological status of the cyanobacterium^25,26^.

To test these hypotheses, we experimentally studied intraspecific interactions using three strains of *N. spumigena* with specific NRP profiles from CT_B (Supplementary Fig. 1)^9^. Of the three strains, two belonged to the same chemotype subgroup (B2), and the third was from a more distant subgroup (B3)^9^. To mimic the co-existence of these strains in the pelagia, we designed a co-culture system, where the test strains were incubated in chambers joined at the bottleneck and separated from each other by a membrane filter. The monocultures were grown in the same way using the same strain in each chamber. These experimental settings allowed us to study the strain-specific reciprocal effects in a continuous exposure with diffusible compounds passing through the membrane without any direct cell-to-cell contact. We measured chlorophyll *a* fluorescence, photosynthetic efficiency, and NRP levels in the strains under monoculture and co-culture conditions to assess their growth, photosynthetic activity, and metabolic response. To our knowledge, this is the first study considering intraspecific interactions in *N. spumigena*, with a particular focus on physiological and metabolic responses.

## Methods

### Experimental setup

A co-culture system that consisted of two modified glass flasks, 500 mL each, fitted together by a holding clamp around their bottlenecks, was built *in-house* (Supplementary Fig. 2). The two compartments were separated by a 0.22-μm hydrophilic polyvinylidene fluoride (PVDF) membrane filter (Durapore, Merck, Darmstadt, Germany) mounted between the bottlenecks; the membrane enabled a free passage for the growth media and dissolved substances but not for the cells. All components of the co-culture system were autoclaved separately and assembled under sterile conditions.

### Diffusion assay

The exchange of dissolved organic compounds between the two chambers of our co-culture system was tested in a pilot study with *N. spumigena* cell extract containing 12 NRPs (three SPUs, one AER, six APs, and two NODs); with a range of atomic mass of 0.58–0.93 kDa; with measurements at 0, 6, and 10 h. The results show that the NRPs were uniformly dispersed in the system after 10 h of incubation (Supplementary Fig. 3 and Supplementary Table 1). Details of the experiment and the results can be found in Supplementary Data 1.

### Strains and culture conditions

Three Baltic *N. spumigena* strains with different NRP profiles, CCNP1403, CCNP1430 and CCNP1440^9^; hereafter referred to as 1403, 1430 and 1440, respectively, were selected from the Culture Collection of Northern Poland, Division of Marine Biotechnology, Gdańsk University, Poland. The strains were non-axenic. All three strains were isolated from the Gulf of Gdańsk and belong to the chemotype cluster CT_B and the subgroups B2 and B3 (Supplementary Fig. 1)^9^. This cluster was selected due to its high chemodiversity, but relatively low number of NRPs. The latitudes/longitudes and year of isolation were the following: 54° 29′ N/18° 40′ E, 1997; 54° 35′ N/18° 47′ E, 2012 and 54° 30′ N/18° 33′ E, 2013 for 1403, 1430, and 1440, respectively^9^. The strains 1403 and 1430 produce the same NRP variants and are grouped in the same chemotype subgroup B2 (Supplementary Fig. 1)^9^. The strain 1440 produces some NRPs that are shared with 1403 and 1430 and NRPs that are structurally different variants of the same classes; thus, 1440 belongs to a different chemotype subgroup, B3 (Supplementary Fig. 1)^9^. The cultures were grown in f/2 medium supplemented with NaCl to reach salinity 7 and maintained in exponential growth phase by a regular dilution with fresh f/2 medium. The temperature was set to 20 ± 2 °C and irradiance to 30 μmol photons m^−2^ s^−1^ with a light:dark cycle of 16:8 h.

### Mono- and co-culture treatments

**Figure 1:**
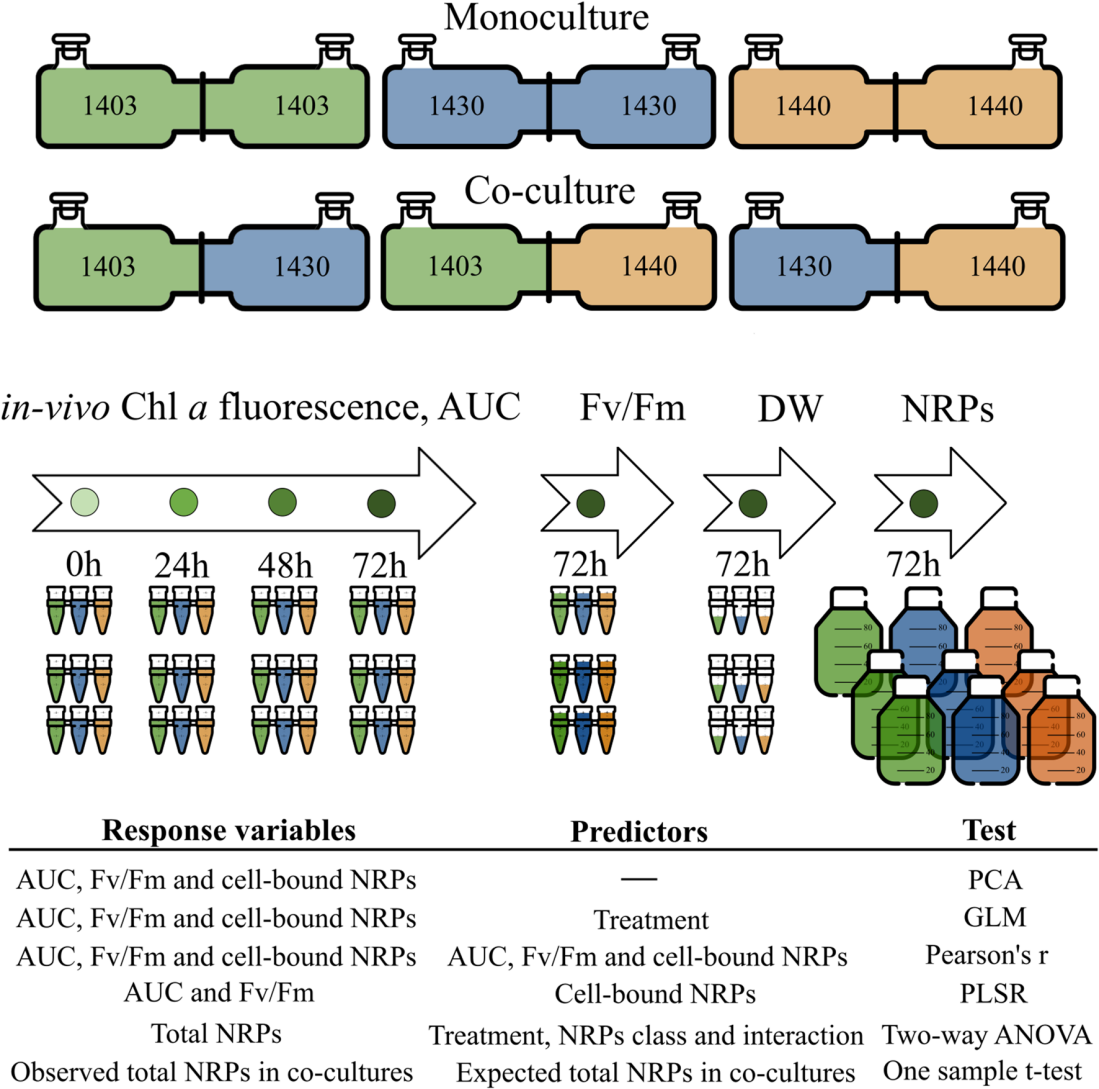
Schematic representation of the experimental design. Each treatment, e.g., monocultures 1403/1403, 1430/1430, 1440/1440 and co-cultures 1403/1430, 1403/1440 and 1430/1440, were performed in triplicate. The initial biomass of the cyanobacterium was the same among the treatments. Samples for growth assessment (area under the growth curve, AUC) were taken as a time series (0 h, 24 h, 48 h and 72 h). Samples for photosystem II maximum yield (Fv/Fm), dry weight (DW), cell-bound and extracellular non-ribosomal peptides (NRPs) were taken at the time point 72 h. Statistical methods included PCA, Principal Components Analysis; GLM, Generalized linear models; Pearson correlation; Spearman's rank correlation; Two-way Anova including post-hoc analysis with Tukey’s HSD test and One sample t-test. This image has been designed using resources from Flaticon.com.

We experimented using different combinations of mono- and co-cultures (Fig. 1). Cultures of all exponentially growing strains were diluted with the f/2 media to the same in-vivo chlorophyll *a* value measured in triplicate using a 10AU Field Fluorometer (Turner Designs, Sunnyvale, California, USA). In the monoculture treatments, each chamber (Fig. 1) was inoculated with the same strain. The co-culture treatment 1403/1430 was started by inoculating one chamber with 1403 and the other with 1430; then, the chambers were joined to enable the exchange of media during the incubation (Fig. 1). The same procedure was applied to the treatments 1403/1440 and 1430/1440. Each co-culture and monoculture treatment were run in triplicate. The experiment lasted 72 h, with all mono- and co-cultures incubated at the same conditions as the inoculum cultures and shaken at approximately 50 rpm.

### Sampling and sample preparation

Samples (3 mL) were taken at time points 0, 24, 48, and 72 h from all culture chambers to determine in-vivo chlorophyll *a* fluorescence. At the time point 72 h, samples (4 mL) were also taken from all chambers for Photosystem II (PSII) efficiency measurements (Fig. 1). Simultaneously, the cyanobacteria were harvested from both chambers by filtration under vacuum (100 mL) onto 47 mm GF/F filters (Whatman, Kent, UK). The wet filters with the cells retained were transferred to 2-mL Eppendorf tubes, dried by rotary evaporation in an Eppendorf Concentrator 5301 (Eppendorf, Hamburg, Germany), and stored at − 80 °C until extraction. The filtrates were concentrated by a solid-phase extraction method using 500 mg Oasis HLB cartridges (Waters, Milford, MA, USA)^9^. The 100% methanol eluate was evaporated to dryness following the same procedure as for the cell-bound NRP samples. The extracts were kept at − 80 °C until further workup. Additionally, 1-mL samples were collected at 72 h from all culture chambers and dried in the Eppendorf Concentrator 5301 at 60 °C. These samples were used to determine cyanobacterial dry weight using a Sartorius BP211D Analytical Balance with a readability of 0.01 mg (Sartorius Lab Instruments GmbH & Co. KG, Goettingen, Germany).

### Physiological parameters

The in-vivo chlorophyll *a* fluorescence was measured in the samples taken at each time point. Chlorophyll *a* fluorescence is a surrogate for algae biomass according to the OECD guideline^27^. As an integral measure of growth during the exposure, we used the area under the curve (AUC) calculated as a change in fluorescence intensity between the measurements. This endpoint is commonly used in algal growth inhibition assays^27^ as it integrates the change in photosynthetically active biomass during the time of exposure^28^.

The maximum quantum yield of PSII (Fv/Fm) is a proxy of physiological state^29,30^. Using a Pulse Amplitude Modulation fluorometer (PAM; Heinz Walz GmbH, Effeltrich, Germany), we measured Fv/Fm in the strains under mono- and co-culture conditions. The instrument consisted of a MODULAR version of the DUAL-PAM-100 measuring system including a Power- and Control-Unit DUAL-C, an Emitter DUAL-E, and DUAL-DR Measuring Head with Detector connected to a PC operated with DUAL PAM v1.19 software. Immediately after collection, the samples were dark-adapted for 20-min. After dark adaptation, Fv/Fm was determined by applying a single saturation pulse. Samples were illuminated for 10 s with actinic light (wavelength 660 nm), and the Fv/Fm values were calculated by the instrument software.

### Cell-bound and extracellular NRPs

#### Extraction

Prior to liquid chromatography–tandem mass spectrometry (LC–MS/MS) analysis, 1 mL 75% methanol in MilliQ water was added to the dried filters (containing the cell-bound NRPs) and to the dried filtrate SPE eluates (potentially containing extracellular NRPs). Filters were macerated with a fine glass rod. Subsequently, the samples (filters and filtrates) were homogenized in an ultrasonic bath (Sonorex, Bandelin, Berlin, Germany) for 5 min and vortexed for another 5 min. Subsequently, the samples were centrifuged for 10 min at 2 °C with 10,000 rpm. After centrifugation, the supernatants were transferred to chromatographic vials and analysed by LC–MS/MS.

#### LC–MS/MS analysis and quantification

LC–MS/MS analyses were performed according to Mazur-Marzec et al*.*^9^. Samples were analysed on an Agilent 1200 (Agilent Technologies, Waldboronn, Germany) coupled to a triple-quadrupole mass spectrometer (5500 QTRAP, AB Sciex, Concord, ON, Canada) using a Zorbax Eclipse XDB-C18 column (4.6 mm × 150 mm; 5 μm; Agilent Technologies, Santa Clara, CA, USA) with a mobile phase composed of 5% acetonitrile in MilliQ water (A) and acetonitrile (B), both containing 0.1% formic acid. The injection volume was 5 μL and the flow rate was 0.6 mL min^−1^. The column temperature was 35 °C. The gradient elution started with 15% of mobile phase B, rising to 50% B over 10 min and then to 99% B in 5 min, held for 10 min, then decreased to 15% B in 2 min and held for 10 min to equilibrate the system. Mass spectrometer operated in positive mode, with turbo ion spray (550 °C) voltage 5.5 kV and declustering potential of 80 V. The information-dependent acquisition method (IDA) was used and fragmentation spectra of all ions with m/z in a range 500–1000 and signal above the threshold of 500,000 cps were collected. The NRP relative concentrations in the extracts were determined based on the peak area of the extracted ion chromatogram. Data acquisition and processing were accomplished using Analyst QS 1.5.1 software (AB Sciex, Concord, ON, Canada). Peak areas of cell-bound NRPs were normalized to the dry mass using the corresponding filter sample at 72 h following the common practice by dividing the peak area with the dry mass^31,32^. When both strains in the co-culture were capable of producing the same NRPs, the extracellular NRPs could not be assigned to a specific strain. Therefore, a uniform approach was applied to all treatments by normalizing peak areas for each NRP detected to the total dry mass of the *N. spumigena* in the system.

### Data analysis and statistics

All statistical analyses, if not stated otherwise, were carried out in JMP Version 14.0. SAS Institute Inc., Cary, NC, 1989–2019; the significance level was set to α = 0.05. The cell-bound NRPs relative concentrations (normalized peak areas), Fv/Fm, and AUC values were used for the statistical comparisons. The extracellular NRPs relative concentrations (normalized peak areas) were used for calculating the total NRP stocks in the system. The primary data on these variables are presented as mean value ± standard error (*n* = 3).

To visualize treatment effect on the cell-bound NRPs in each strain, the relative concentrations of NRPs were expressed as the fold change for their normalized peak areas in the co-cultures to that in the monocultures. The fold change was calculated as $\frac{A}{B}$; with A being a replicate of a co-culture NRP peak area, and B—the average monoculture NRP peak area.

First, Principal Components Analysis (PCA) on covariances was conducted to (1) explore the overall variability between the treatments, i.e., three monocultures and their co-cultures (1403/1430, 1403/1440, and 1430/1440); and (2) examine the relationships between the metabolic (normalized cell-bound NRPs) and physiological (Fv/Fm and AUC) variables of the test strains. The normalized cell-bound NRPs were used as response variables, and Fv/Fm and AUC values as supplementary variables. The explained proportion of the variance and the minimum number of the components were determined by the broken stick model (Supplementary Fig. 4). Pearson's correlation analysis was applied to further explore cross-correlations among the cell-bound NRPs in each strain across the treatments.

Second, Generalized Linear Models (GLM) were used to evaluate whether the treatment as a categorical predictor (co-culture *vs.* monoculture) significantly affected the cell-bound NRPs, AUC values, and Fv/Fm values; this analysis addressed hypothesis H1. The cell-bound NRP values were log-transformed, whereas no data transformation was needed for AUC and Fv/Fm values. Model performance and homoscedasticity were confirmed by the residual and Q-Q plot analyses. The GLMs with normal error distribution and identity link-function were analyzed using the *glm* function from package *stats* v3.6.2 in R Studio 3.6.3^33^.

To evaluate if NRP levels are positively related to the physiological state of the cyanobacterium (H4), a spearman rank correlation between the physiological parameters (AUC and Fv/Fm values) and cell-bound NRPs measured in the mono- and co-cultures was performed.

To compare the total (cell-bound and extracellular) NRP concentrations in the system between the monocultures and co-cultures, the cumulative NRP values were normalized to either the dry mass of the cyanobacterium in the system or the total volume. The individual NRPs were grouped by class, i.e., SPUs, NODs, APs, and AERs, and the final values were log-transformed. A two-way ANOVA with treatment (monoculture *vs.* co-culture), NRP class (SPU, NOD, AP, and AER), and *treatment* × *NRP* interaction was applied to each monoculture/co-culture data set. The data were centered to enable interpretation of the main factors when the interaction term was significant. We used Tukey’s HSD test for multiple comparisons to evaluate the treatment effect for each NRP class.

Finally, we compared the expected NRP quantities to those observed in the co-culture using the constant yield expectation model (ConYE) introduced by Medlock et al.^34^. The ConYE model, assumes that each strain produces or consumes a fixed quantity of each metabolite per unit biomass (i.e., constant yield). By comparing the expected and the observed concentrations in the co-culture, one can identify potential metabolic interactions that contribute to growth modulation in co-culture. The expected NRP quantities in the co-cultures were simulated by multiplying the monoculture-derived NRP concentrations for each strain by the observed abundance of that strain in the co-culture and summing up the obtained values to estimate the total NRP quantity in the system. The observed NRP values in each co-culture (1403/1430, 1403/1440, and 1430/1440) were compared to their expected values using a one-sample t-test; this comparison addressed hypothesis H1.

## Results

### Growth and photosynthetic efficiency

**Figure 2:**
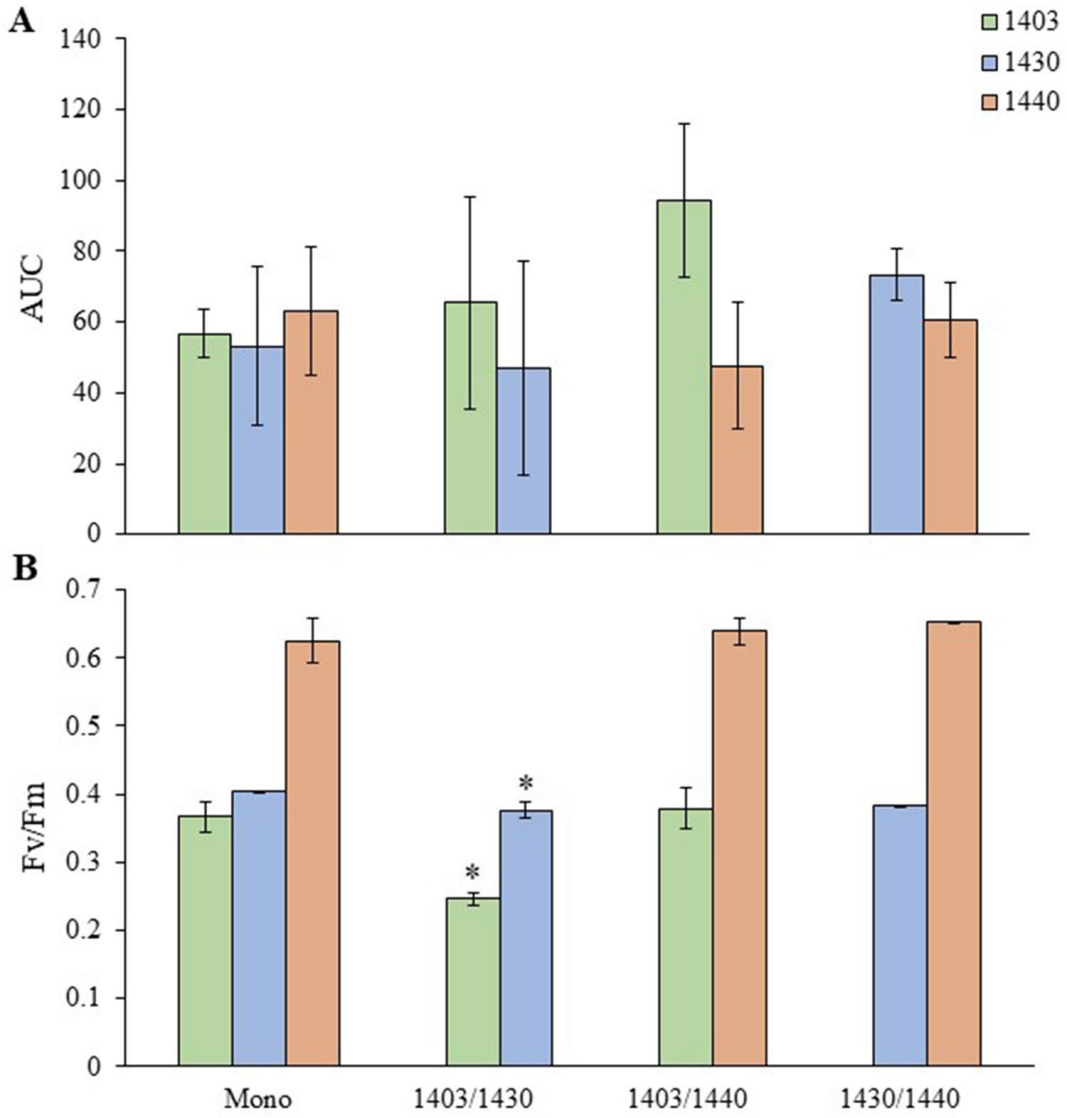
Growth and photosynthesis measured as (**A**), Area under the curve (AUC); and (**B**), Maximum quantum yield of PSII (Fv/Fm) for *N. spumigena* strains 1403, 1430, and 1440 grown as a monoculture and co-cultures (1403/1430, 1403/1440, and 1430/1440). All data are shown as mean ± standard error (*n* = 3); asterisk indicates significant (*p* < 0.05) difference from the monoculture of the respective strain based on the GLM output (Supplementary Table 4).

In all *N. spumigena* strains, the mean growth (AUC) was similar between the mono- and co-cultures with no significant growth response to the co-culturing (Fig. 2A and Supplementary Table 4). In 1403 and 1430, the Fv/Fm values were significantly lower in the co-cultures (1403/1430) than in their respective monocultures (Fig. 2B and Supplementary Table 4). No significant difference was detected for the Fv/Fm values between the co-cultures (1403/1440 and 1430/1440) and the respective monocultures (Supplementary Table 4).

### Cell-bound NRP levels

**Figure 3:**
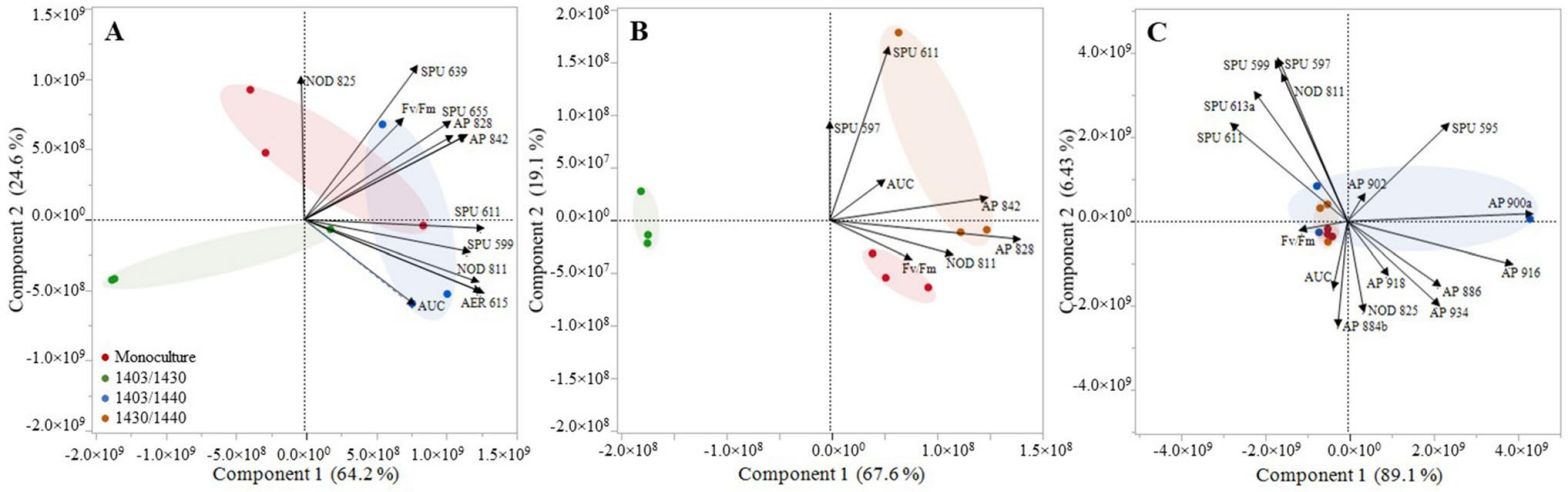
Principal Component Analysis (PCA plots showing Component 1 and Component 2 with the variance explained) of the cell-bound NRP levels in *N. spumigena*; (**A**), 1403; (**B**), 1430; and (**C**), 1440. Treatments: monoculture (red) and co-cultures: 1403/1430 (green), 1403/1440 (blue) and 1430/1440 (orange); NRP classes are denoted as: SPU, spumigins; NOD, nodularins; AER, aeruginosins and AP, anabaenopeptins.

The PCA identified two significant PCs based on the NRP levels for strain 1403 and one PC for strains 1430 and 1440 (Supplementary Fig. 4). The PCA biplots suggested some separation between the mono- and co-culture treatments. For strain 1403, a clear separation was observed for the co-culture 1403/1430, whereas there was some overlap between the monoculture and co-culture 1403/1440 (Fig. 3A). For strain 1430, a separation between all treatments was found (Fig. 3B). By contrast, there was no clear separation between the treatments for 1440 (Fig. 3C).

For strain 1403, four SPUs (SPU 597, SPU 611, SPU 639, and SPU 655), AER 615, and NOD 825 had the highest loadings (≥ 0.3), and, therefore, the greatest influence on the grouping in PC1 and PC2, which together explained 89% of the variance (Supplementary Tables 2 and 3). For strain 1430, the two SPUs (SPU 597 and SPU 599) and the two APs (AP 828 and AP 842) had the highest loadings, explaining 87% of the variance (Supplementary Tables 2 and 3). For strain 1440, PC1 explained 89% of the variance, with the highest loading (0.98) for AP 900a (Supplementary Tables 2 and 3).

The PCA biplots also suggested some cross-correlations between the NRPs. For strain 1403, SPU 597, SPU 599, SPU 611 as well as for AP 828 and SPU 641 were changing in concert (Fig. 3A).

In line with the PCA patterns, significant correlations (Pearson's *r* > 0.7) across all treatments for strain 1403 between (1) SPU 597, SPU 599, SPU 611, AER 615 and NOD 811; (2) AP 828, AP 842, SPU 611, SPU 639, SPU 641; and (3) AP 842 and SPU 655, were found (Supplementary Fig. 5A). For strain 1430, the eigenvectors of AP 842, AP 828 and NOD 811 had a similar ordination (Fig. 3B); accordingly, these three NRPs were significantly positive correlated across all treatments (Supplementary Fig. 5B). For strain 1440, several NRP vectors had similar ordinations (Fig. 3C), and the SPUs (SPU 597, SPU 599, SPU 611, and SPU 613a) were significantly correlated with each other as well as with NOD 811 (Supplementary Fig. 5C). Additional correlations were found among the APs (AP 884b, AP 886, AP 918 and AP 934), between NOD 825 and APs (AP 884b, AP 886, AP 902, AP 916 and AP 934) (Supplementary Fig. 5C).

**Figure 4:**
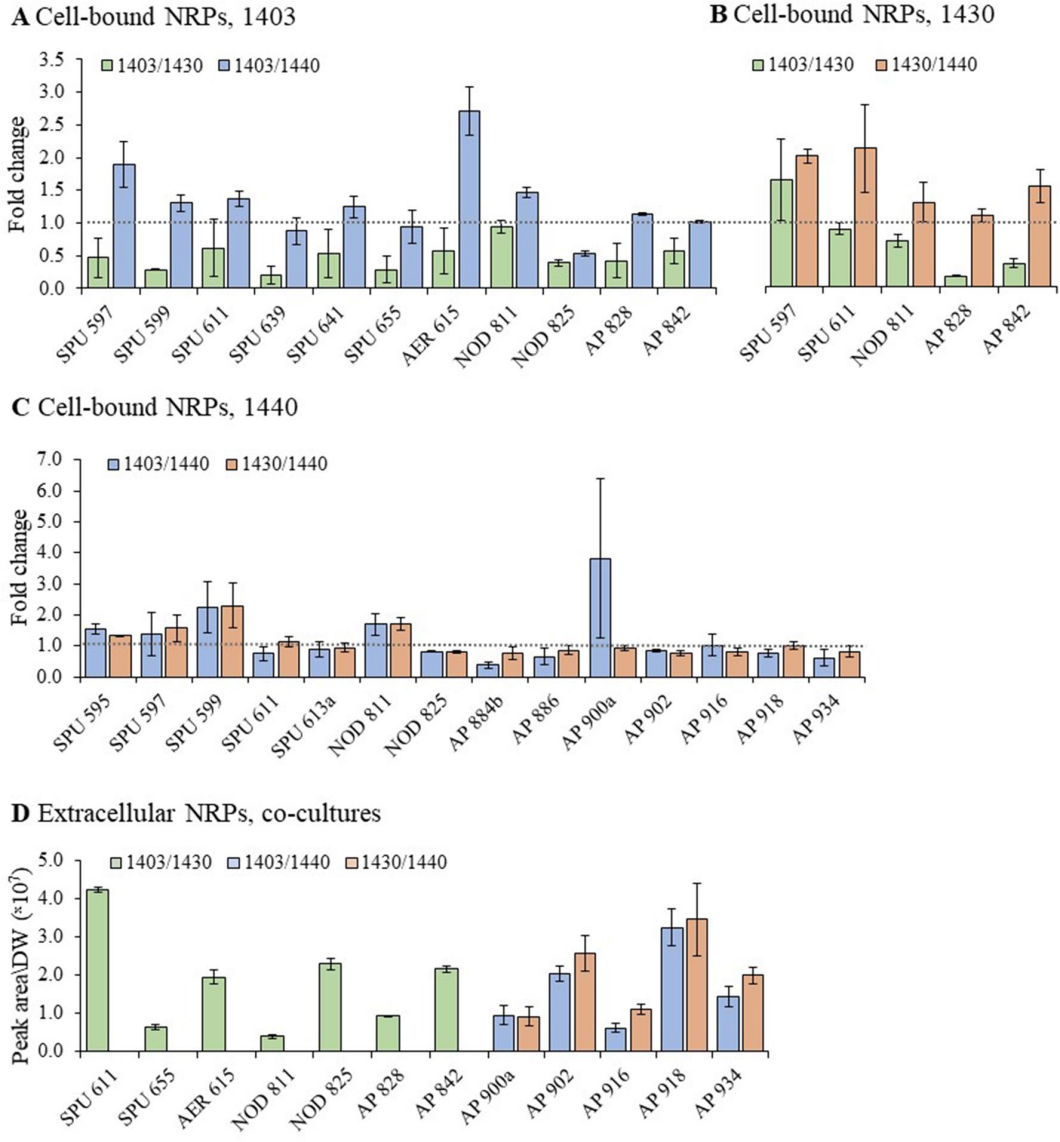
Relative concentrations of (**A**), 1403; (**B**), 1430; (**C**), 1440 cell-bound NRPs and (**D**) extracellular NRPs in co-cultures. Relative concentrations of cell-bound NRPs are given as a fold change over *N. spumigena* respective monocultures (mean ± standard error, *n* = 3). Grey dash-line represents onefold change, i.e. concentrations equal to monoculture. Relative concentrations of extracellular NRPs in co-cultures are given as dry weight normalized peak areas (mean ± standard error, *n* = 3). Note that no extracellular NRPs were detectable in monocultures of the same strains. Treatments: co-cultures 1403/1430 (green), 1403/1440 (blue) and 1430/1440 (orange). NRP classes are denoted as SPU, spumigins; NOD, nodularins; AER, aeruginosins and AP, anabaenopeptins.

When strains 1403 and 1430 were co-cultured (1403/1430), most NRPs were twofold lower than in the monocultures. By contrast, in the co-cultures with 1440 (1403/1440 and 1430/1440), the concentrations for most NRPs were up to threefold higher (Fig. 4A,B). For strain 1440, the differences between the co-culture treatments (1403/1440 and 1430/1440) and monoculture were minor (Fig. 4C). However, in both co-cultures, SPU 599, NOD 811, and AP 900a had concentrations 2- to fourfold higher than in the monocultures (Fig. 4C).

The GLMs revealed significant differences for some but not all NRPs between the *N. spumigena* co-cultures and the respective monocultures (Supplementary Table 5). For example, in 1403, the concentration of SPU 599, SPU 639 and SPU 655 were significantly lower in the 1403/1430 co-culture than the monoculture, whereas NOD 811 was significantly higher in the 1403/1440 co-culture (Supplementary Table 5). In 1430, the concentrations of AP 828 and AP 842 were significantly lower in the co-culture 1403/1430 than the monoculture (Supplementary Table 4). In 1440, NOD 825 was significantly lower in both co-cultures compared to the monoculture, and in the co-culture 1403/1440, levels of AP 884b and SPU 595 were significantly higher and lower, respectively (Supplementary Table 5).

### Extracellular NRP levels

No extracellular NRPs were detected in any *N. spumigena* monocultures, whereas some were found in all co-cultures (Fig. 4D). The highest extracellular NRP diversity was detected in the co-culture 1403/1430, where seven NRPs from all classes were found (Fig. 4D). Except for SPU 655 and AER 615 belonging to the strain 1403 profile, all these NRPs can be produced by both 1403 and 1430. In 1403/1430, the extracellular NRPs contribution for the total pool of NRPs (per DW) varied between 7% (NOD 811) and 29% (SPU 597). Interestingly, none of the extracellular NRPs were detected in the co-cultures with 1440. Instead, five APs (AP 900a, AP 902, AP 916, AP 918, and AP 934) were detected in the co-cultures 1403/1440 and 1430/1440 (Fig. 4D). These APs are produced by strain 1440 but not by the other strains (Fig. 4); therefore, their levels can be attributed exclusively to the response by 1440 exposed to the other strains. Their contribution to the total pool of NRPs (per DW) in the co-cultures varied between 0.33% (AP 900a) and 3.76% (AP 918) for 1403/1440 and between 1.29% (AP 900a) and 3.89% (AP 902) for 1430/1440.

### Correlations between physiological variables and NRPs

**Figure 5:**
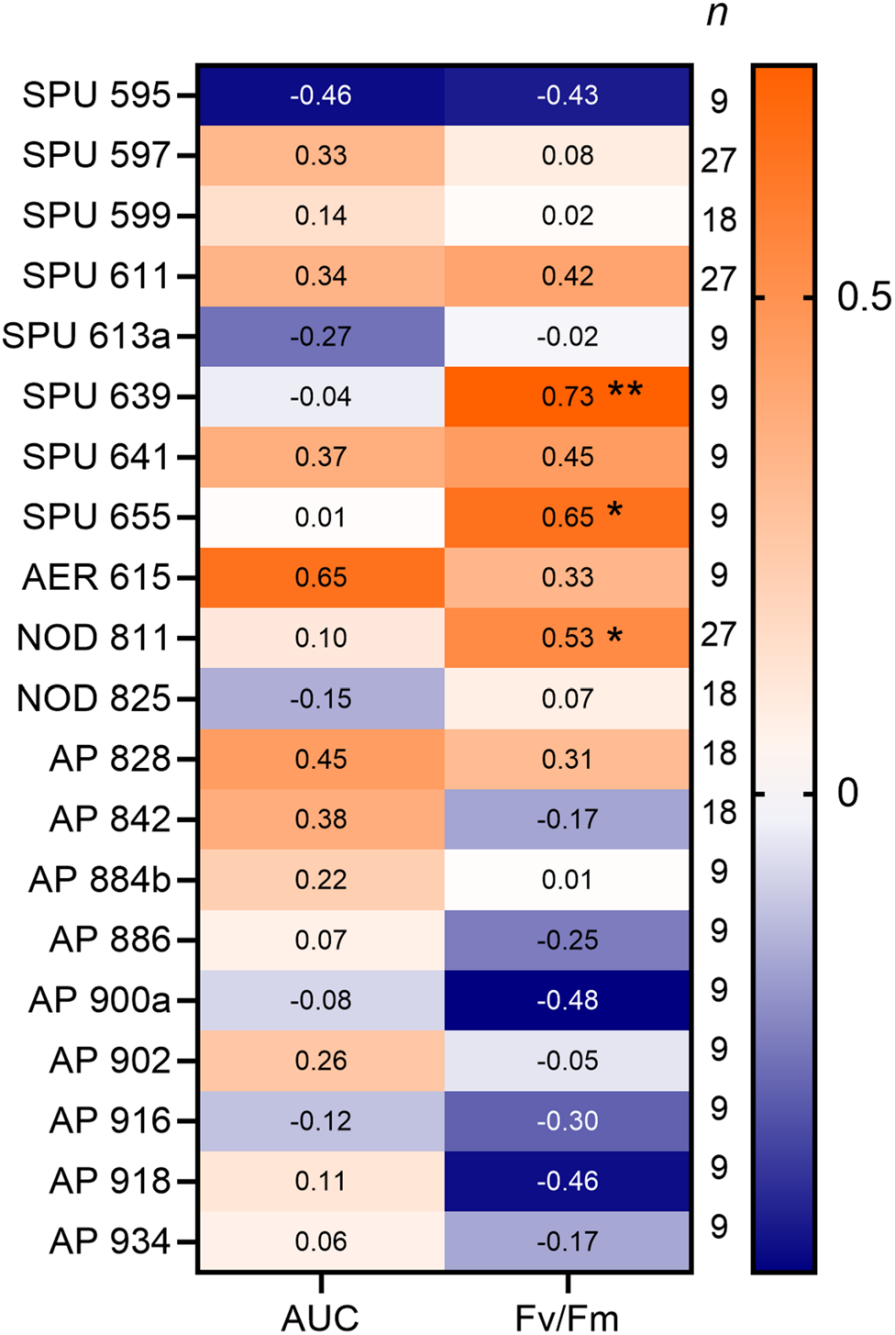
Spearman rank correlations between the physiological parameters (AUC: area under the curve, representing the cumulative biomass; Fv/Fm: photosynthetic efficiency) and NRPs measured in the mono- and co-cultures, all strains, and treatments included. The numbers in the cells are rho values, and the asterisks denote *p* < 0.05 (*) and *p* < 0.01 (**); *n* values indicate number of observations.

There were significant positive correlations between the Fv/Fm values and three NRPs (Fig. 5): SPU 639 and SPU 655 (strain 1403) and NOD 811 (all strains combined). No significant correlations between AUC values and any of the NRPs were found.

### Total NRP quantities in mono- vs. co-cultures

**Figure 6:**
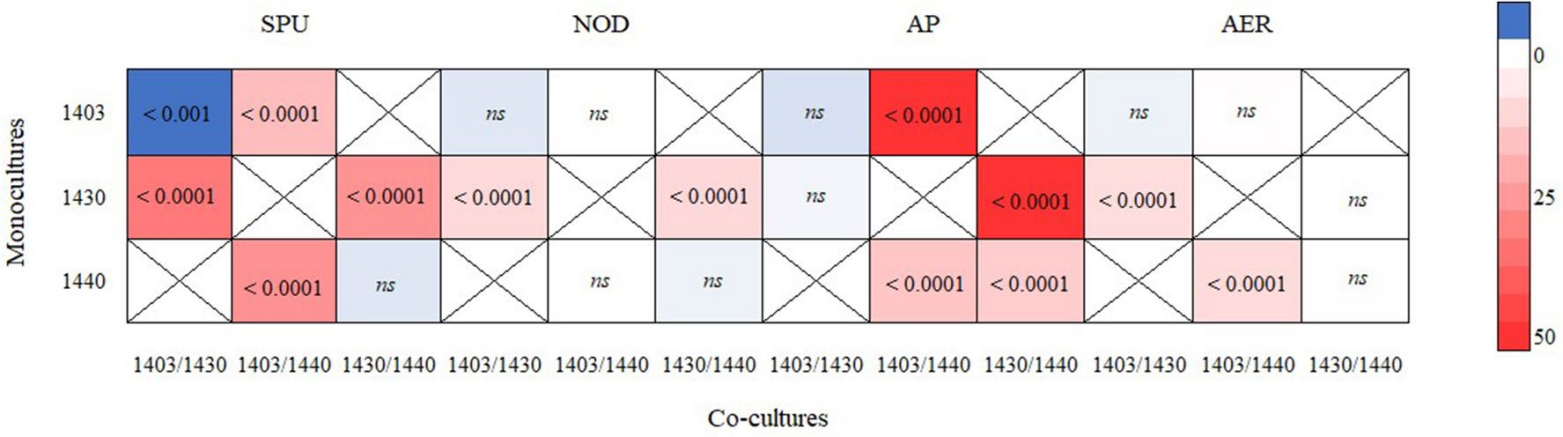
Results of post-hoc with Tukey’s honestly significant difference (HSD) on the effect of treatment on each NRP class concentration; see Supplementary Table 6 for details. The cell-plot colors represent the mean difference between the mono- and co-cultures, with blue showing higher values in monoculture and red in co-culture. NRP classes are: SPU, spumigins; NOD, nodularins; AER, aeruginosins and AP, anabaenopeptins. The *p*-value of the test result indicated in each cell; *ns* non-significant.

The total NRP levels, i.e., a sum of cell-bound and the extracellular fractions, in the *N. spumigena* co-cultures were not significantly different from the monocultures, except 1403 vs. 1403/1430 (Supplementary Fig. 6). In strain 1403, all individual NRPs tended to be somewhat higher in the monoculture than in 1403/1430 (Supplementary Fig. 6). When NRPs were grouped by class, significantly higher values were observed for the co-cultures in the most pair-wise comparisons (Supplementary Table 6). In 1403 vs. 1403/1430 the *treatment* × *NRP* interaction was significant because of the SPU class, which was significantly higher in the mono- than in the co-culture, whereas all other NRP classes were not significantly different between 1403 and 1403/1430 (Supplementary Table 6 and Fig. 6).

### Observed vs. expected NRP production in co-cultures

**Figure 7:**
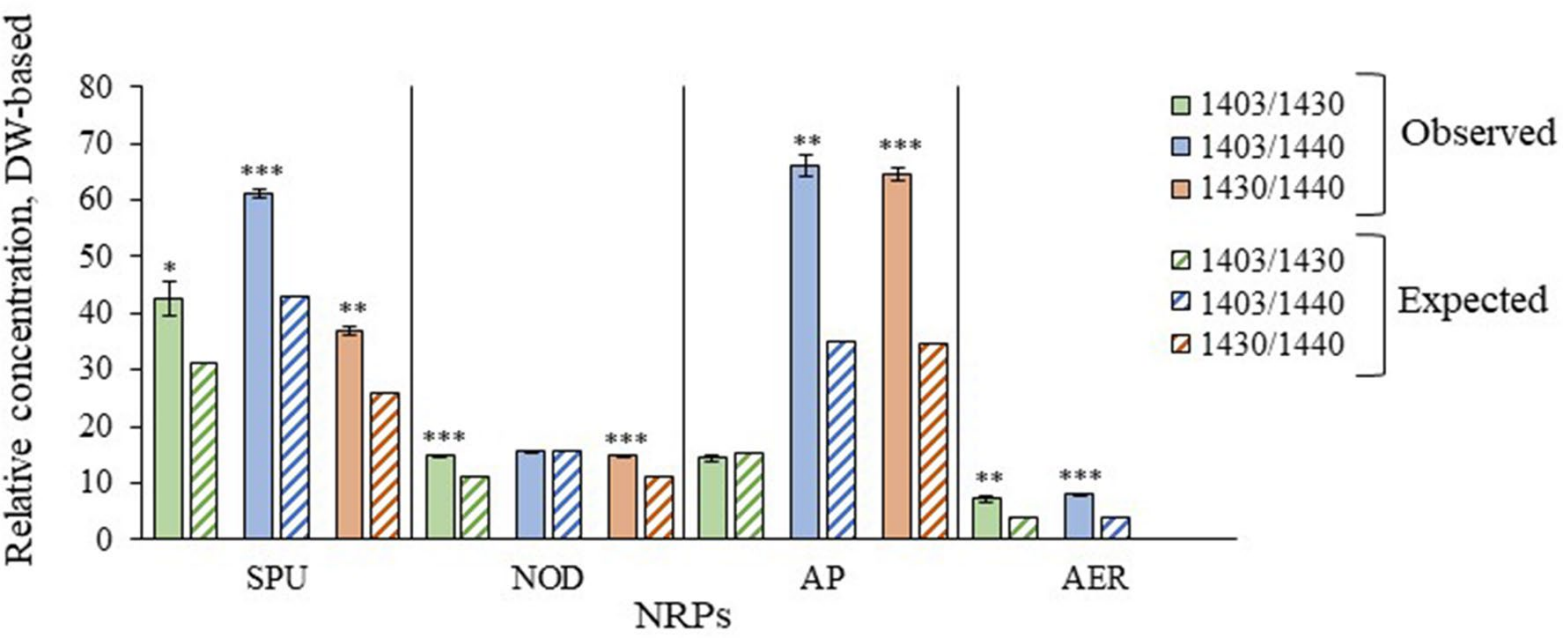
Observed and expected concentrations of total NRPs in *N. spumigena* co-cultures according to the ConYE model. Total NRPs were pooled by class. NRP classes are denoted as SPU, spumigins; NOD, nodularins; AER, aeruginosins and AP, anabaenopeptins. Only results of dry-weight normalized NRPs concentrations are presented; volume-based results were omitted from the figure due to their similarity with DW-based results. For each NRP class, the significant difference between observed and expected concentrations was tested with one sample t test; *, **, and *** indicates *p-*value < 0.05, 0.01, and 0.001, respectively.

For all NRP classes, the observed total concentrations in the *N. spumigena* co-cultures were, in most cases, significantly higher than the expected concentrations estimated using the NRP production under monoculture conditions. The only exceptions were NOD and AP classes in 1403/1440 and 1403/1430, respectively, where there were no significant differences between the observed and the expected values (Fig. 7).

## Discussion

### Co-culture setup

Our co-cultivation system allowed different *N. spumigena* strains to be physically separated while sharing a common culture medium and interacting via chemical signalling. Thus, the observed physiological and metabolic responses can be attributed to these interactions. The pilot assay showed that the NRPs added to one compartment diffused readily (Supplementary Table 1). The use of analogous co-culture systems has been shown to facilitate studies on chemical communication between planktonic organisms^31,35,36^. Other designs using dialysis bags or cellophane tubing placed in a glass pot were less advantageous because of higher uncertainty due to possible differences in the growth conditions or inadequate diffusion between the system compartments^37–40^. Another advantage of our setup over commercially available double chamber co-culture systems^41^ is the larger culture volume (up to 500 mL) that allows sampling for metabolomic investigations even in dilute cultures that resemble the cyanobacteria population density in the environment^42^. Thus, the co-culture approach is useful because it mimics ecological settings, where secondary metabolites may mediate intra- and interspecific interactions.

### Co-culturing induced physiological and metabolic responses

Our findings partially support hypothesis H1 predicting physiological and metabolic differences between the *N. spumigena* monocultures and co-cultures. When strains 1403 and 1430 were co-cultured, the photosynthetic efficiency was significantly reduced in both strains compared to their monocultures (Fig. 2B and Supplementary Table 4), whereas no photosynthesis alterations were found in the other co-cultures. Moreover, no significant treatment effect on cyanobacterial growth (AUC) was found for any co-cultures (Fig. 2A and Supplementary Table 4). It is possible that the observed response in the photosynthetic efficiency was insufficient to induce a measurable growth response at the duration/light/nutrient conditions used in our experiment. Moreover, the uncoupled photosynthesis efficiency and growth may manifest costs for production or breakdown of extracellular metabolites^43^, which might have occurred in our system. It is also possible that in-vivo chlorophyll *a* fluorescence was not a sufficiently sensitive indicator of cyanobacteria biomass affecting AUC-based growth assessment. Using organic carbon and cell number could have provided a more robust biomass indicator^44,45^.

For all strains, we found significant differences in the cell-bound NRP levels between the co-cultures and the respective monocultures (H1; Supplementary Table 5). A similar experiment aimed to evaluate whether intraspecific interactions regulated NRP production in *Microcystis aeruginosa*^31^. In the co-culture of a microcystin (MC)-producing strain and an MC‐deficient mutant of the same strain (PCC 7806), the NRP levels were significantly higher than in the monoculture for both strains, but the growth rate was unaltered. By contrast, a natural non-MC-producing strain (PCC 9432) co-cultured with the MC-producing strain had a significantly higher growth rate and NRP levels than in monoculture, whereas the growth and NRP levels of the MC-producing strain were unaffected^31^. For *M. aeruginosa*, it has been shown that NRP profiles could be clustered primarily according to their global metabolite content, then according to their genotype, and finally according to their sampling location, i.e., variation in biotic and abiotic factors in the environment^11^. Our findings support these conclusions and show that some strains of *N. spumigena* have more labile NRP profiles than others and are more likely to respond to other strains in the population. Considering that *N. spumigena* cultures were non-axenic, the associated microbiome of the cyanobacteria might also contribute to the intraspecific interactions and NRPs levels. The heterotrophic bacteria associated with *N. spumigena* have been shown to provide the cyanobacteria cells with vitamin B12, CO_2_, and regenerated nutrients, assist in organic matter decomposition and removal of toxic metals as well as degrading complex organic molecules, such as NRPs produced by the cyanobacteria^46–50^.

### Chemotype-related responses

Strains with stronger responses to the co-culturing (treatment 1403/1430) belong to the same chemotype subgroup, whereas strain 1440 is from a more distinct chemotype subgroup. Therefore, these findings support hypothesis H2 and suggest that in *N. spumigena* intraspecific competition between closely related strains is more likely. However, considering that we used only three strains from the chemotype clusters CT_B and no strain of CT_A, it remains to be tested whether this holds true for the entire *N. spumigena* population. In line with the photosynthesis efficiency response, the changes in the cell-bound NRPs were more pronounced in the 1403/1430 treatment (Fig. 3). Moreover, in this treatment, both strains had twofold lower levels for most cell-bound NRPs (all five classes, i.e., SPUs, AERs, NODs, and APs), whereas extracellular NRPs comprised approximately 21% of the total pool of NRPs. By contrast, in the co-cultures with strain 1440, the NRP concentrations were up to threefold higher than in the monocultures (Fig. 4A,B); with no NRP produced by 1403 and 1430 detected extracellularly.

As hypothesized (H3), strain 1440 responded similarly to co-culturing with strains 1403 and 1430 with nearly identical levels of both cell-bound and extracellular NRPs in 1403/1440 and 1430/1440 (Fig. 4C). The appearance of extracellular APs in these treatments coincided with lower levels of the cell-bound APs (Fig. 4C,D). Thus, strain 1440 responded similarly to the co-culturing with 1403 and 1430 by releasing APs to the media. Also, supporting H3, strains 1403 and 1430 responded similarly to co-culturing with 1440 (Fig. 4A,B,D).

In line with our results, the co-culture of two *M. aeruginosa* strains with similar NRP profiles (i.e. *M. aeruginosa* PCC 7806 and its MC‐deficient mutant) led to the production of higher NRP levels than their respective monocultures, and the co-cultures between two *M. aeruginosa* strains with different NRP profiles (i.e. *M. aeruginosa* PCC 7806 and PCC 9432)^31,51^. Also, *Planktothrix agardhii* exposed to two distinct cell extracts of bloom samples predominated by *P. agardii* (95% total biomass) had its NRPs levels both higher or lower than in the controls (monocultures), depending on the cell extract^52^. It is important to note that each cell extract consists of *P. agardii* strains of several chemotypes of different NRP profiles^52,53^. Therefore, it can be concluded that cyanobacteria intraspecific interactions promote a more chemical diverse population; co-existing strains of more distinct chemotypes produce more NRPs than co-existing strains of similar chemotypes.

### Linkages between NRP production and physiological status

Penn et al*.*^54^ detected transcripts for PKS/NRPS (enzymes involved in NRPs synthesis) genes from *Microcystis* strains throughout the day/night cycle, suggesting that these genes are continually expressed. Thus, NRPs are likely central to cell physiology rather than produced in response to transient environmental cues. The NRPs are currently considered secondary metabolites; that is, they rarely have a role in primary metabolism, growth, or reproduction but have evolved to benefit the cyanobacterium^55^. Nevertheless, the biosynthesis of NRPs requires significant cellular energy and nutrient resources. Any single amino acid incorporated in an NRP requires about 4–5 kbp of genetic information, and, although the share of NRPs synthetase enzymes in the cellular protein pool is unknown, it has been assumed that the translation of the enzymes is energetically costly^12^. NRPs are derived mainly from primary metabolites, which are modified by i.e. methylation, glycosylation, epimerization, or halogenation, thus, NRPs share common precursors with growth, while photosynthesis provides a resource (the shared primary carbon) for all these physiological pathways. Accordingly, higher NRP concentrations have been associated with optimal growth conditions^25,26^. In *Microcystis aeruginosa*, a positive relationship between the microcystin content of cells and their specific growth rate was reported^56^. Additionally, several other physiological functions have been suggested for cyanotoxins (including microcystin), such as photosynthesis efficiency, carbohydrate metabolism, cell signaling, nutrient uptake, iron scavenging, maintenance of homeostasis, and protection against oxidative stress^22,57–61^.

It has been known that secondary metabolites in plants are intimately involved with primary metabolic functions^62^. However, very few studies of cyanobacteria have considered such functions of NRPs, including cyanotoxins. Some of microcystin, for example, binds covalently to proteins in a light conditions and depending on iron availability^63^. Moreover, cell redox state rather than the light intensity has been shown to be more relevant for the conjugate formation^63,64^. Such reports support the suggested role of microcystin, and potentially other NRPs, in primary metabolism, functioning as a protein-modulating metabolites and protectants against oxidative stress, growth and cell division in cyanobacteria^65^. Notably, protection from the oxidative stress has been advocated as the reason for NRP production by ancestral cyanobacteria^63,64,66^. Moreover, several studies have shown that cyanotoxins (some of them NRPs) production by cyanobacteria is largely unaffected by environmental stressors and that cyanotoxins are produced in nearly fixed amounts during cell division^65,67–69^. Although in cyanobacteria, the cell quota (i.e., average amount of metabolite per cell) are relatively invariant for each strain, they may vary by several orders of magnitude between the strains^65,69^.

We found that photosynthetic efficiency (Fv/Fm) was positively correlated with the cell-bound SPU 639 and SPU 655 in strain 1403 and with NOD 811 in all strains combined. No significant correlations between NRPs and AUC values were found. Hence, there might be a functional potential for NRPs to be involved in energy acquisition and metabolism. The uncoupling of growth and photosynthesis may also reduce the photosynthetic performance of cyanobacteria through a decline in enzymes of photosynthetic carbon metabolism and, consequently, in cellular photosynthetic capacity^70,71^.

### NRPs may have complementary functions or interlinked pathways

Significant strong positive cross-correlations were found for some NRPs in different strains across the treatments (Supplementary Fig. 5). Notably, the negative correlations were much less common than positive correlations, with no strong negative correlations observed. Therefore, one can speculate that there were no antagonistic relations between the NRPs analysed. For NRPs of the same class, e.g., SPUs, NODs, and APs, the strongest positive cross-correlations might be explained by a commonality of the metabolic pathways^16,72,73^. For instance, SPU 611, SPU 599, and SPU 597, have strong positive cross-correlations in the strains 1403 and 1440 (Supplementary Fig. 5A,C). Structurally, SPU 611 comprises a hydroxyphenyl lactic acid (Hpla) in the N-terminal position, argininal in C-terminus, and (2S, 4S)-4-methylproline (MePro) in position 3. The only difference between SPU 599 and SPU 611 is the change of MePro for Pro, and between SPU 599 and SPU 597 is the alteration of argininal for argininol^16,74^. Significant correlations were also detected between APs, for instance, AP 828 and AP 842, that have largely the same chemical structure, except that AP 842 has isoleucine instead of valine^9^. Based on the structural similarities, AERs and SPUs are anticipated to be related compounds^15,75^; however, these two NRP classes are assembled by different peptide synthetases^16^. Additionally, biosynthesis of SPUs, AERs, and APs proceeds on large multienzyme complexes, non-ribosomal peptide synthetases (NRPS), composed of several modules^14–16^, while NODs are synthesized by a hybrid non-ribosomal peptide and polyketide pathway^72^. Therefore, the correlations between NRPs from different classes, particularly with NODs, cannot be related to the interlinked metabolic pathways. These correlations indicate that some NRPs may have complementary functions in the cyanobacterium, as previously suggested^31,52^. Further studies on the mechanisms behind the synchronized dynamics of such NRPs are needed.

### Role of extracellular NRPs

*Nodularia spumigena* is well known to cause both inhibition and stimulation of growth^76–78^ as well as inhibition of photosynthesis in green algae, diatoms, cryptomonads, and other cyanobacteria^79^. Similarly, several other cyanobacterial genera (*Anabaena*; *Calothrix*; *Gomphosphaeria*; *Aphanizomenon*; *Hapalosiphon*; *Fischerella*; *Microcystis*; *Nostoc*; *Oscillatoria*; *Phormidium*; *Scytonema*; and *Trichormus*) have been described to engage in interspecific interactions; moreover, some allelochemicals or potential allelochemicals involved in these interactions have been identified^80,81^. These allelochemicals include chlorinated aromatic compounds, cyclic and non-cyclic peptides, polyketides, alkaloids, and fatty acids^82^. Although specific allelochemical agents are still unknown for *N. spumigena*, NOD, being structurally closely related to the known allelochemical microcystin^83,84^, has been suggested to confer this function. Other NRP classes have also been suggested as possible allelochemical agents^12,31,76^. However, if the primary function of NRPs is to exert allelopathic effects, they must be actively excreted by the cyanobacterium and not remain cell-bound until released during the cell lysis at the breakdown of the bloom as suggested in the literature^85–87^.

In line with that, the extracellular NRPs were detected in all co-cultures but not in the monocultures. Moreover, the release of extracellular NRPs coincided with a decrease in their cell-bound concentrations. Given that co-culturing induced no growth response, there is no indication that the NRP release was associated with the cell lysis in our experiment. Thus, extracellular NRPs alone or in concert with other (unmeasured) allelochemicals could be responsible for the observed responses in photosynthesis and cell-bound NRPs. In *M. aeruginosa*, the experimental addition of purified microcystin, micropeptin or microginin, enhanced expression of the *mcy* gene cluster and microcystin production^88^, and downregulated secondary metabolite gene expression^89^. In our study, extracellular APs produced by strain 1440 were detected in 1403/1440 and 1430/1440, and the total AP levels were significantly higher in the co-culture than in any monocultures. Extracellular SPUs produced by both strain 1403 and 1430 were detected in 1403/1430, and the total SPU levels were significantly higher in the co-culture than in 1430 monoculture. Thus, extracellular NRPs released in response to co-culturing may participate in cell‐to‐cell communication, both as signalling molecules and bioactive metabolites affecting competitors even at the intrapopulation level. Such multiple functions may differ between the intracellular matrix and the surrounding media; as proposed for the NRP class of microcystins^63,88–90^. A subsequent study on *N. spumigena* peptidome responses to exposure to pure single compound or mixtures of NRPs would allow to corroborate our hypothesis and better understand NRPs involvement in allelopathic interactions.

### Ecological advantages of chemical diversity

To have a chemically diverse population is advantageous for cyanobacteria because it may provide (1) diversity of allelopathic metabolites to outcompete other autotrophs^82,91^, (2) more combinations of defensive and deterring compounds suppressing grazing^54,92–94^, and (3) broader resistance against rapidly evolving parasites (chytrid fungi, cyanophages)^95^. Indeed, the Baltic Sea *N. spumigena* population has a high chemodiversity of NRP variants^9^, which may contribute to this cyanobacterium successful development.

In our study, the reduction of photosynthetic efficiency and cell-bound NRP levels in the co-cultured 1403 with concomitant appearance of extracellular NRPs suggest that intraspecific interactions promoted NRP production, at least, in some *N. spumigena* strains. When we applied the ConYE model, the observed total NRP concentrations were significantly higher than the concentrations expected. The significant NRP increase in the co-cultures implies that at least one strain altered its NRP metabolism relative to the biomass production. This increase in concentration further suggests an involvement of NRPs to intraspecific interactions, lending further support to hypothesis H1. However, if the cellular NRP quota is relatively constant, estimates for NRP concentrations based on the cell number might be lower compared to our values based on the dry weight normalization, if the cell mass in the rapidly growing strains are lower^65,96,97^. Of course, *N. spumigena* strains in mixed populations might have different competitive capacities as illustrated by reports that this cyanobacterium can exert both growth stimulation and inhibition in algae^76–79^. More specifically, Lei et al*.*^98^ showed that the allelopathic effect of *Microcystis aeruginosa* towards *Raphidiopsis raciborskii* was strain-dependent. Therefore, using single-strain *N. spumigena* cultures in studies on allelopathy will not help understand the *in-situ* effects of *N. spumigena* population composed of coexistent strains.

## Conclusions

In co-existing strains of *N. spumigena*, the production of NRPs and physiological status can be mediated by the extracellular NRPs or other potential allelochemicals produced by *N. spumigena*. Moreover, strains from the same chemotype subgroup respond similarly to co-culture treatments, increasing their NRPs levels when co-cultured with a strain from other chemotype and reducing it when co-cultured with a same-subgroup strain. Positive correlations between the NRPs from different classes indicate coordinated responses to the culture conditions and suggest that NRPs may have complementary functions. Correlations of some SPUs in 1403, and NOD 811 in all *N. spumigena* strains with photosynthetic efficiency indicates a possible involvement of these NRPs in cell physiology. Moreover, the release of extracellular NRPs into the media in response to co-culturing supports their cell-to-cell communication/allelopathy functions. Overall, the interactions involving the NRP production and the associated linkages to the physiological performance would promote chemical diversity in a population, with a concomitant increase in NRP production, dissolved metabolite concentrations, and possible food-web effects.

## Supplementary Information

Supplementary Information.
