# Supplementary material for: Competitive interactions as a mechanism for chemical diversity maintenance in Nodularia spumigena
Source: Sci Rep. 2021 Apr 26;11:8970. doi: 10.1038/s41598-021-88361-x (PMC8076297; doi:10.1038/s41598-021-88361-x)
Supplement: Supplementary file 1 — Supplementary Information. [file 41598_2021_88361_MOESM1_ESM.pdf]

Supplementary Information

Competitive interactions as a mechanism for chemical diversity maintenance in *Nodularia spumigena*

Sandra Lage[1,2;*], Hanna Mazur-Marzec[2], Elena Gorokhova[1]

[1] Department of Environmental Science, Stockholm University, Sweden

[2] Division of Marine Biotechnology, Institute of Oceanography, University of Gdańsk, Poland

[*]Correspondence: Sandra.Lage@aces.su.se

Contents

Page

Supplementary Data 1. Pilot tests. Diffusion assay with *N. spumigena* cell extract. 3

Supplementary Figure 1. Cluster analyses of peptide profiles detected in *N. spumigena* from the Baltic Sea. 5

Supplementary Figure 2. Photograph of co-culture system. 6

Supplementary Figure 3. Ratio of the NRP peak areas between the *Donor* and the *Receiver* compartments. 7

Supplementary Figure 4. Scree plot of proportion of variance explained by principal components. 8

Supplementary Figure 5. Pearson's cross-correlations between the cell-bound NRPs in *N. spumigena*. 9

Supplementary Figure 6. Total concentrations of NRPs in *N. spumigena* monocultures and co-cultures. 10

Supplementary Table 1. Results of the one-sample t-test for the ratio of the NRP peak areas between the *Donor* and the *Receiver* compartments in the pilot experiment. 11

Supplementary Table 2. Principal Components (PC), eigenvalues and % of variance, and cumulative % of variance explained by the PCs. 12

Supplementary Table 3. Eigenvectors of Principal components 1 and 2. 13

Supplementary Table 4. GLM output for the models testing treatment effects on the physiological variables (Fv/Fm and AUC) in the experiments with *N. spumigena.* 14

Supplementary Table 5. GLM output for the models testing treatment effects on the log-transformed cell-bound NRPs in the experiments with *N. spumigena.* 15

Supplementary Table 6. Outcome of two-way ANOVAs testing treatment effects on total NRPs concentrations of *N. spumigena.* 17

References 18

Supplementary Data 1. Pilot test

Diffusion assay with *N. spumigena* cell extract

To evaluate if our *in-house* co-culture system (Supplementary Figure 2) allows sufficient diffusion of cyanobacterial metabolites through a 0.22 µm hydrophilic polyvinylidene fluoride (PVDF) membrane filter between both culturing compartments, we performed a diffusion assay with a *N. spumigena* cell extract. The two compartments of the co-culture system were filled with 300 mL of f/2 medium, and one compartment was supplemented with the *N. spumigena* cell extract containing 12 non-ribosomal peptides (NRPs), three spumigins (SPUs), one aeruginosin (AER), six anabaenopeptins (APs), and two nodularins (NODs). To account for potential NRPs losses due to degradation of the NRPs, we also included controls. The controls were run in 500 mL Erlenmeyer flasks filled with 300 mL of sterile f/2 medium and supplemented with the *N. spumigena* cell extract. The two-chamber setups and controls were kept at 20 ± 2 °C, irradiance of 30 µmol photons m^{-2}. s^{-1} and shaken at approximately 50 rpm. To follow the kinetics of diffusion through the membrane, 50 mL samples were taken in duplicates from both compartments and controls at time points 6 and 10 h. Samples were also taken at time 0h. Following, the samples were concentrated by solid phase extraction method using 500 mg Oasis HLB cartridges (Waters, Milford, MA, USA)[1]. The elutes were evaporated to dryness by rotary evaporation in an Eppendorf concentrator 5301 (Eppendorf, Hamburg, Germany), and stored at −80 °C until analysis. The membranes of the co-culture system were also analysed in the end of the experiment, to evaluate NRPs sorption to the membranes. LC-MS/MS analyses and were performed according to Mazur-Marzec, *et al.* [1].

First, using the measured NRP for each experimental unit, we calculated a ratio of the NRP concentrations between the *Donor* and the *Receiver* compartments *(Donor/Receiver* ratio) as a measure of the concentration difference. There was no significant time effect (6 h vs. 10 h) on the *Donor/Receiver* ratio across the NRPs (Paired t-test; $t_{11} = 1.445$; $p > 0.17$). Hence, the samples collected at both time points were pooled to evaluate whether the NRP concentrations were significantly different between the compartments after the 10-h incubation.

If the *Donor/Receiver* ratio is significantly higher than 1, this would indicate a lack of equilibrium (Supplementary Figure 3). We used one-sample t-test to compare the *Donor/Receiver*

ratio with a theoretical mean of 1; four samples (two from each time point) were used for each NRP. In no case, the discrepancy from 1 was significant (Supplementary Table 1), although for NOD 811 and AP 852 it was relatively high and marginally significant. Thus, we can conclude that after 10 h of the incubation, the NRP concentrations were similar between the compartments, suggesting efficient diffusion and suitability of the system for the experiment. Considering that the main experiment was run over 72 h, we are rather confident that all NRPs had sufficient time to distribute evenly in the system.

Previously, dissolved organic compounds of around 0.1 kDa have been shown to significantly diffuse through a 0.22 µm PVDF membrane between two chambers within the first hours after addition[2]. Diffusion of the Microcystin-LR standard (0.99 kDa), an NRP with a molecular weight equivalent NRPs produced by *N. spumigena*, through a 0.45 µm cellulose nitrate membrane in co-culture setup reached equilibrium between the two compartments within 48 h[3]. It must be noted that diffusion between co-culture setups might be affected by the pore size, diameter and material of the membrane used.

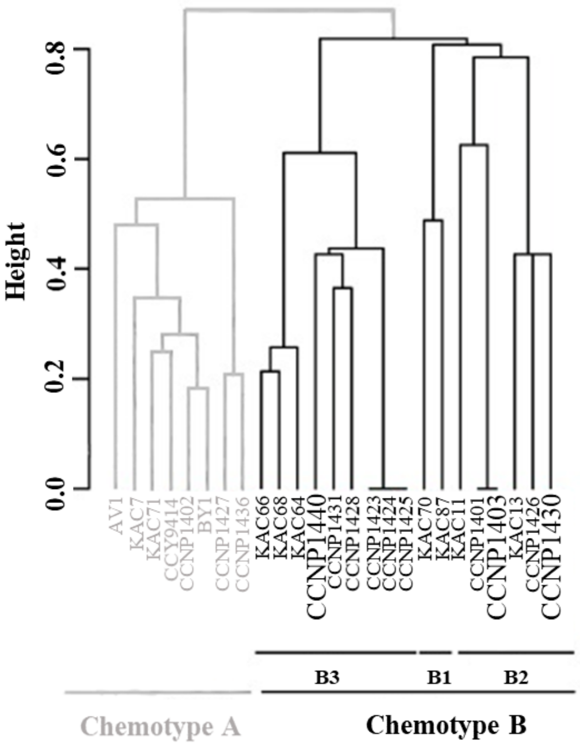

Supplementary Figure 1. Cluster analyses of non-ribosomal peptides profiles detected in *N. spumigena* from the Baltic Sea (adapted from Mazur-Marzec, *et al.* [1]). Two chemotype clusters were identified CT_A and CT_B. The strains clustered in CT_A have the highest number of NRPs (22–35), the highest frequency of pseudoaeruginosins, and all APs are nodulapeptins (i.e. contained methionine or serine/acetylserine). CT_B is more heterogenous than CT_A; it comprises strains with a higher chemodiversity, but with a lower number of NRPs (from 10 to 22) than strains from CT_A. Within CT_B, three subgroups (B1, B2 and B3) differing in some components were established. The strains in B1 have APs with isoleucine in the exocyclic position and have the same sequence of residues. While the strains of B2 have both isoleucine and phenylalanine in the position 1 of APs. Additionally, in the SPUs produced by B2 strains it was observed a modification in *N*-terminal Hpla residue (Hpla + 42), and the presence of leucine and tyrosine in position 2. The strains in B3 have APs with the same three-amino acid sequence (phenylalanine, isoleucine and valine)[1]. Only strains of CT_B isolated from the Gulf of Gdańsk were used in this study, i.e. CCNP1403, CCNP1430 and CCNP1440.

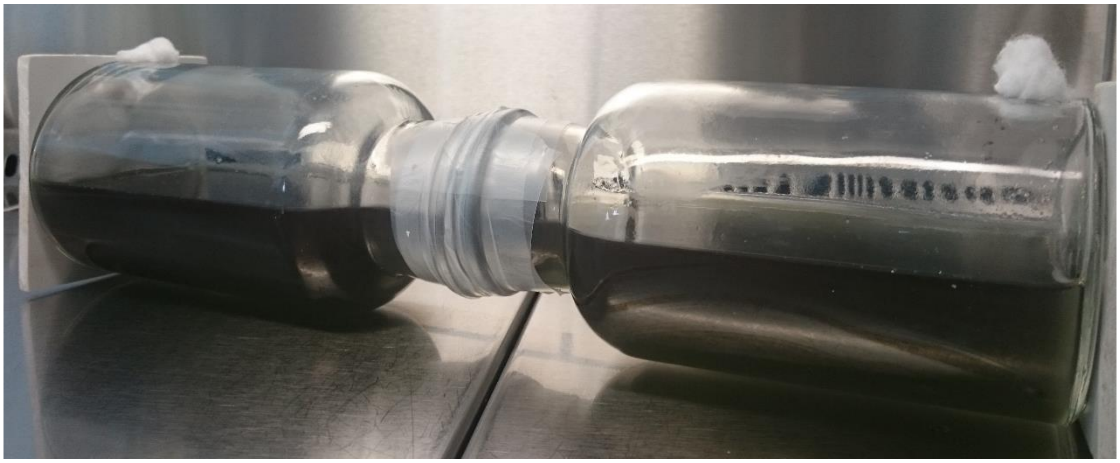

Supplementary Figure 2. Photograph of the co-culture system, consisting of two modified glass flasks each holding 500 mL fitted together by a holding clamp. The two chambers are divided by a 0.22 μm hydrophilic polyvinylidene fluoride (PVDF) membrane filter, that enabled the passage of solutions and dissolved substances, but not cells. All components of the co-culture system were autoclaved separately and assembled under sterile conditions. The photo was taken by Sandra Lage.

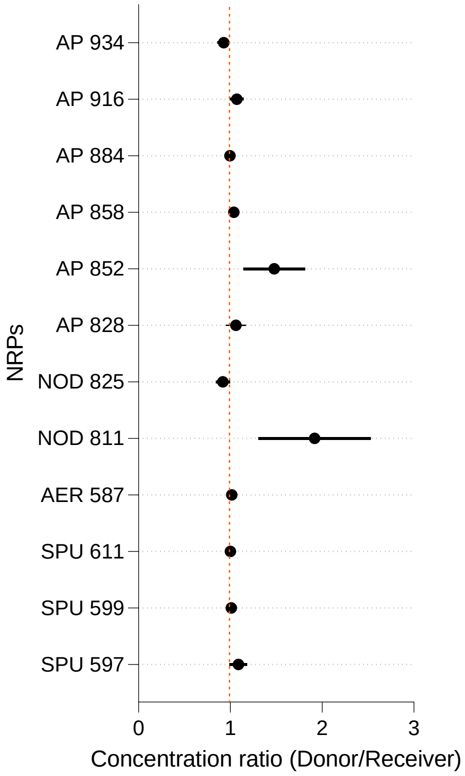

Supplementary Figure 3. Observed ratio of the NRP peak areas between the *Donor* and the *Receiver* compartments in the pilot experiment; the data are shown as mean ± SD; n =4. The vertical line indicates the *Donor/Receiver* ratio = 1 corresponding to the equal concentrations between the compartments. See Supplementary Table 1 for the results of the one-sample t-test evaluating deviation from the ratio =1 ($p > 0.05$ in all cases).

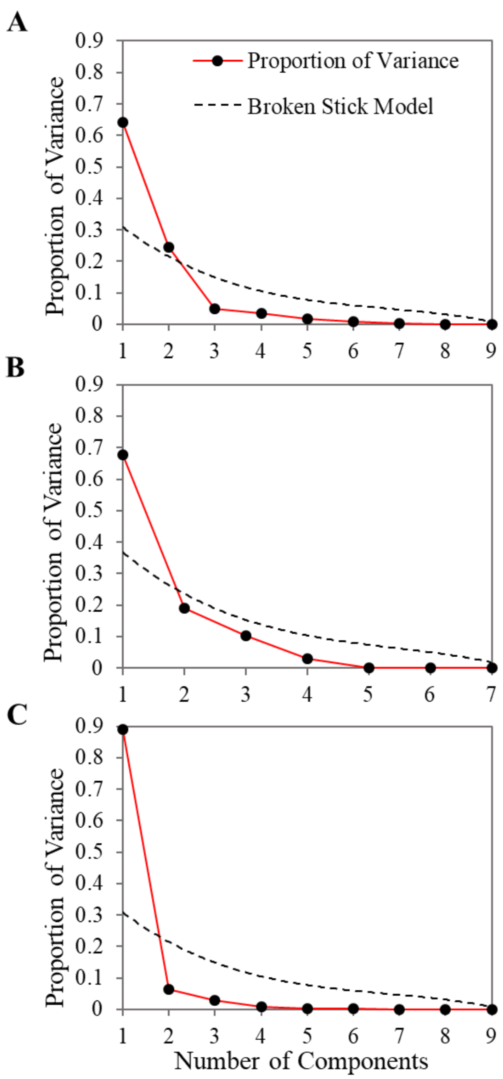

Supplementary Figure 4. Scree plots of the proportion of variance explained by principal components for the metabolic (intracellular NRPs abundance and diversity) and physiologic (Fv/Fm and AUC) variables in the experiments with *N. spumigena* strains: (A), 1403; (B), 1430; and (C), 1440. The dashed curves indicate the expected proportions that result from a broken stick model. Significance of the components was obtained by comparing explained proportion of the variance (red line) and the minimum proportion of the variance expectation under the broken stick model (dashed line).

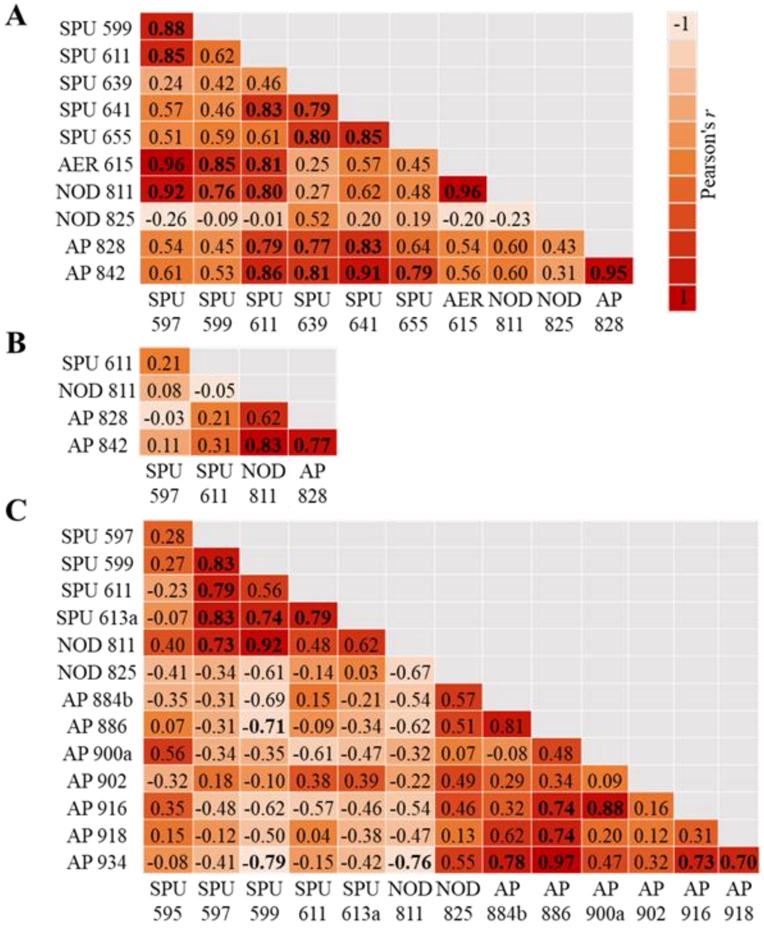

Supplementary Figure 5. Pearson's cross-correlations between the cell-bound NRPs in *N. spumigena*; (A), 1403 (treatments: monoculture 1403, 1403/1430 and 1403/1440); (B), 1430 (treatments: monoculture 1430, 1403/1430 and 1430/1440), and (C), 1440 (treatments: monoculture 1440, 1403/1440 and 1430/1440). The color intensity reflects the correlation coefficients value (r) as indicated by the scale bar. Strong positive correlations ($r > 0.7$) are indicated in bold.

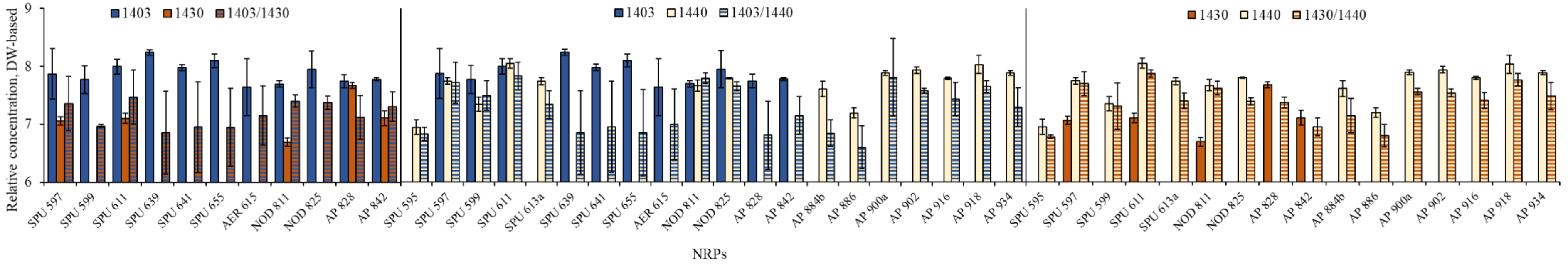

Supplementary Figure 6. Total concentrations of NRPs in *N. spumigena* monocultures and co-cultures. Each value indicates sum of cell-bound and extracellular NRPs; mean ± standard error, n = 3. NRP classes are denoted as SPU, spumigins; NOD, nodularins; AER, aeruginosins; AP, anabaenopeptins. Only results of dry weight normalized NRPs concentrations are presented; volume-based results were omitted from the figure due to their similarity with DW-based results.

Supplementary Table 1. Results of the one-sample t-test for the ratio of the NRP peak areas between the *Donor* and the *Receiver* compartments in the pilot experiment. The discrepancy values represent the difference in the ratio between the actual mean and the theoretical mean value of 1 when the concentrations between the compartments are equal. Each data set consisted of four samples.

NRP	t, df	*p* value	Discrepancy	SD of discrepancy	95% confidence interval
SPU 597	1.839, 3	0.163	0.088	0.096	-0.064 to 0.240
SPU 599	1.000, 3	0.391	0.010	0.020	-0.022 to 0.043
SPU 611	0.042, 3	0.969	0.001	0.031	-0.049 to 0.051
AER 587	2.428, 3	0.094	0.017	0.014	-0.005 to 0.041
NOD 811	2.988, 3	0.059	0.918	0.614	-0.060 to 1.896
NOD 825	2.113, 3	0.125	-0.081	0.076	-0.202 to 0.040
AP 828	1.121, 3	0.344	0.061	0.110	-0.113 to 0.236
AP 852	2.851, 3	0.065	0.478	0.335	-0.055 to 1.011
AP 858	2.658, 3	0.076	0.039	0.030	-0.008 to 0.086
AP 884	1.000, 3	0.391	-0.004	0.008	-0.017 to 0.009
AP 916	1.909, 3	0.152	0.071	0.074	-0.047 to 0.189
AP 934	2.158, 3	0.119	-0.072	0.067	-0.178 to 0.034

Supplementary Table 2. Principal Components (PC), eigenvalues and % of variance, and cumulative % of variance explained by the PCs of the PCA of the metabolic (cell-bound NRP levels) and physiologic (Fv/Fm and AUC) variables in the experiments with *N. spumigena* strains 1403, 1430 and 1440.

Strains	PC	Eigenvalue	% Variance	Cumulative % Variance
**1403**	1	8.29×10^{17}	64.21	64.21
	2	3.17×10^{17}	24.56	88.76
	3	6.36×10^{16}	4.93	93.69
	4	4.52×10^{16}	3.51	97.20
	5	2.15×10^{16}	1.66	98.86
	6	1.18×10^{16}	0.92	99.77
	7	2.76×10^{15}	0.21	99.99
	8	1.47×10^{14}	0.01	100.00
	9	49.15	0.00	100.00
**1430**	1	1.84×10^{16}	67.62	67.62
	2	5.18×10^{15}	19.08	86.70
	3	2.75×10^{15}	10.13	96.83
	4	8.29×10^{14}	3.05	99.89
	5	3.11×10^{13}	0.11	100.00
	6	717.20	0	100.00
	7	1.25	0	100.00
**1440**	1	2.65×10^{18}	89.10	89.10
	2	1.91×10^{17}	6.43	95.53
	3	8.97×10^{16}	3.02	98.55
	4	2.21×10^{16}	0.75	99.30
	5	8.37×10^{15}	0.28	99.58
	6	7.05×10^{15}	0.24	99.81
	7	3.62×10^{15}	0.12	99.94
	8	1.9×10^{15}	0.06	100.00
	9	67.98	0.00	100.00

Supplementary Table 3. Eigenvectors of Principal components 1 and 2 (PC1 and PC2) of the PCA metabolic (cell-bound NRP levels) and physiological (Fv/Fm and AUC) variables in the experiments with *N. spumigena* strains 1403, 1430 and 1440.

Strains	Variables	PC1	PC2
1403	SPU 597	0.55	-0.36
	SPU 599	0.20	-0.06
	SPU 611	0.36	-0.03
	SPU 639	0.30	0.65
	SPU 641	0.27	0.23
	SPU 655	0.31	0.34
	AER 615	0.49	-0.32
	NOD 811	0.08	-0.05
	NOD 825	0.00	0.39
	AP 828	0.12	0.11
	AP 842	0.10	0.08
	Fv/Fm	0.00	0.00
	AUC	0.00	0.00
1430	SPU 597	0.00	0.39
	SPU 611	0.16	0.90
	NOD 811	0.06	-0.03
	AP 828	0.94	-0.18
	AP 842	0.29	0.08
	Fv/Fm	0.00	0.00
	AUC	0.00	0.00
1440	SPU 595	0.01	0.02
	SPU 597	-0.08	0.69
	SPU 599	-0.05	0.41
	SPU 611	-0.10	0.32
	SPU 613a	-0.04	0.19
	NOD 811	-0.04	0.33
	NOD 825	0.00	-0.05
	AP 884b	0.00	-0.14
	AP 886	0.01	-0.03
	AP 900a	0.98	0.15
	AP 902	0.00	0.03
	AP 916	0.09	-0.09
	AP 918	0.03	-0.13
	AP 934	0.06	-0.20
	Fv/Fm	0.00	0.00
	AUC	0.00	0.00

Supplementary Table 4. GLM output for the models testing treatment effects on the physiological variables (Fv/Fm and AUC) in the experiments with *N. spumigena* strains 1403, 1430 and 1440; coefficient estimate (Estimate), standard error (Std. Error), t value and *p*-value of the t-test are shown. Significant effects are in bold.

Strain	Variable	Treatment	Estimate	Std. Error	$t_{2,6}$	*p*
1403	AUC	1403/1430	8.63	36.06	0.24	0.82
		1403/1440	37.56	36.06	1.04	0.34
	Fv/Fm	1403/1430	-0.12	0.03	-3.75	<**0.0001**
		1403/1440	0.01	0.03	0.40	0.71
1430	AUC	1403/1430	-6.16	31.31	-0.20	0.85
		1430/1440	20.20	31.31	0.65	0.54
	Fv/Fm	1403/1430	-0.03	0.01	-2.73	**<0.05**
		1430/1440	-0.02	0.01	-2.00	0.09
1440	AUC	1403/1440	-15.42	22.53	-0.68	0.52
		1430/1440	-2.54	22.53	-0.11	0.91
	Fv/Fm	1403/1440	0.01	0.03	0.43	0.69
		1430/1440	0.03	0.03	0.87	0.42

Supplementary Table 5. GLM output for the models testing treatment effects on the log-transformed cell-bound NRPs in the experiments with *N. spumigena* strains 1403, 1430 and 1440; coefficient estimate (Estimate), standard error (Std. Error), t value and *p*-value of the t-test are shown. Significant effects are in bold.

Strain	Variable	Treatment	Estimate	Std. Error	$t_{2,6}$	*p*-value
1403	SPU597	1403/1430	- 0.53	0.38	-1.39	0.22
		1403/1440	0.39	0.38	1.01	0.35
	SPU 599	1403/1430	-0.49	0.13	-3.86	**<0.01**
		1403/1440	0.16	0.13	1.26	0.26
	SPU 611	1403/1430	-0.63	0.37	-1.73	0.14
		1403/1440	0.14	0.37	0.40	0.71
	SPU 639	1403/1430	-1.08	0.34	-3.13	**<0.05**
		1403/1440	-0.09	0.34	-0.26	0.80
	SPU 641	1403/1430	-0.72	0.37	-1.97	0.10
		1403/1440	0.09	0.37	0.24	0.82
	SPU 655	1403/1430	-0.94	0.38	-2.47	**<0.05**
		1403/1440	-0.09	0.38	-0.23	0.83
	AER 615	1403/1430	-0.33	0.37	-0.90	0.41
		1403/1440	0.61	0.37	1.65	0.15
	NOD 811	1403/1430	-0.03	0.06	-0.51	0.63
		1403/1440	0.17	0.06	2.96	**<0.05**
	NOD 825	1403/1430	-0.34	0.16	-2.09	0.08
		1403/1440	-0.20	0.16	-1.23	0.27
	AP 828	1403/1430	-0.63	0.28	-2.30	0.06
		1403/1440	0.06	0.28	0.23	0.82
	AP 842	1403/1430	-0.32	0.15	-2.16	0.07
		1403/1440	0.01	0.15	0.06	0.96
1430	SPU 597	1403/1430	0.13	0.16	0.79	0.46
		1430/1440	0.30	0.16	1.88	0.11
	SPU 611	1403/1430	-0.05	0.14	-0.39	0.71
		1430/1440	0.27	0.14	1.96	0.10
	NOD 811	1403/1430	-0.16	0.12	-1.36	0.22
		1430/1440	0.08	0.12	0.72	0.50
	AP 828	1403/1430	-0.78	0.05	-16.08	**<0.00001**
		1430/1440	0.03	0.05	0.70	0.51
	AP 842	1403/1430	-0.46	0.13	-3.60	**<0.05**
		1430/1440	0.19	0.13	1.46	0.19
1440	SPU 595	1403/1440	0.19	0.08	2.37	**<0.05**
		1430/1440	0.13	0.08	1.67	0.14
	SPU 597	1403/1440	-0.02	0.26	-0.09	0.93

	1430/1440	0.14	0.26	0.53	0.61
SPU 599	1403/1440	0.26	0.28	0.94	0.39
	1430/1440	0.27	0.28	0.96	0.37
SPU 611	1403/1440	-0.18	0.15	-1.20	0.27
	1430/1440	0.04	0.15	0.28	0.79
SPU 613a	1403/1440	-0.09	0.13	-0.69	0.51
	1430/1440	-0.04	0.13	-0.27	0.79
NOD 811	1403/1440	0.20	0.11	1.77	0.13
	1430/1440	0.22	0.11	1.97	0.10
NOD 825	1403/1440	-0.09	0.03	-3.18	**<0.05**
	1430/1440	-0.10	0.03	-3.78	**<0.01**
AP 884b	1403/1440	-0.46	0.19	-2.40	**<0.05**
	1430/1440	-0.16	0.19	-0.84	0.43
AP 886	1403/1440	-0.29	0.20	-1.40	0.21
	1430/1440	-0.09	0.20	-0.44	0.68
AP 900a	1403/1440	0.21	0.33	0.65	0.54
	1430/1440	-0.04	0.33	-0.11	0.91
AP 902	1403/1440	-0.08	0.06	-1.25	0.26
	1430/1440	-0.13	0.06	-2.13	0.08
AP 916	1403/1440	-0.06	0.15	-0.41	0.70
	1430/1440	-0.10	0.15	-0.67	0.53
AP 918	1403/1440	-0.11	0.11	-0.98	0.37
	1430/1440	0.01	0.11	0.05	0.96
AP 934	1403/1440	-0.33	0.22	-1.54	0.17
	1430/1440	-0.13	0.22	-0.61	0.56

Supplementary Table 6. Outcome of two-way ANOVAs testing treatment effects on total NRPs concentrations of *N. spumigena* 1403, 1430 and 1440. NRPs concentrations were summed by class; spumigins, nodularins, anabaenopeptins and aeruginosins. Only results of dry weight normalized NRPs concentrations are presented; volume-based results were omitted from table due to their similarity with DW-based results. Monoculture was selected as reference category. Degrees of freedom (df), Sum-of-squares (SS), F ratio, *p*-value and coefficient estimate (Estimate) are shown. In all two-way ANOVAs the *Treatment* ^{x} *NRPs* interaction was significant, then treatment effect was evaluated for each NRP class using Tukey's HSD test; the post-hoc results are presented in Fig. 6.

			**SS**	**F ratio**	***p*-value**	**Estimate**
Monoculture vs co-culture relative concentrations, DW-based	1403 vs 1403/1430	Treatment	21.26	15.58	< 0.01	- 0.94
		NRPs	6602.97	1612.31	< 0.0001	-
		Treatment ^{x} NRPs	22.58	5.51	< 0.01	-
	1430 vs 1403/1430	Treatment	934.63	770.40	< 0.0001	6.24
		NRPs	2599.51	714.25	< 0.0001	-
		Treatment ^{x} NRPs	908.17	249.53	< 0.0001	-
	1403 vs 1403/1440	Treatment	2111.18	2768.25	< 0.0001	9.38
		NRPs	11186.45	4887.93	< 0.0001	-
		Treatment ^{x} NRPs	3435.31	1501.06	< 0.0001	-
	1440 vs 1403/1440	Treatment	941.36	1504.50	< 0.0001	6.26
		NRPs	17167.99	9146.01	< 0.0001	-
		Treatment ^{x} NRPs	560.52	298.61	< 0.0001	-
	1430 vs 1430/1440	Treatment	3284.73	12935.89	< 0.0001	11.70
		NRPs	7184.11	9430.78	< 0.0001	-
		Treatment ^{x} NRPs	2897.78	3804.00	< 0.0001	-
	1440 vs 1430/1440	Treatment	47.02	177.99	< 0.0001	1.40
		NRPs	16117.98	19994.14	< 0.0001	-
		Treatment ^{x} NRPs	188.54	233.88	< 0.0001	-

df $_{\text{Treatment}}$ = 1, df $_{\text{NRPs}}$ = 3, df $_{\text{Treatment}\ ^{x}\ \text{NRPs}}$ = 3

References

1 Mazur-Marzec, H., Bertos-Fortis, M., Toruńska-Sitarz, A., Fidor, A. & Legrand, C. Chemical and Genetic Diversity of *Nodularia spumigena* from the Baltic Sea. *Mar. Drugs* **14**, 209, doi:10.3390/md14110209 (2016).

2 Paul, C., Mausz, M. A. & Pohnert, G. A co-culturing/metabolomics approach to investigate chemically mediated interactions of planktonic organisms reveals influence of bacteria on diatom metabolism. *Metabolomics* **9**, 349-359, doi:10.1007/s11306-012-0453-1 (2013).

3 Briand, E., Bormans, M., Gugger, M., Dorrestein, P. C. & Gerwick, W. H. Changes in secondary metabolic profiles of *Microcystis aeruginosa* strains in response to intraspecific interactions. *Environ. Microbiol.* **18**, 384-400, doi:10.1111/1462-2920.12904 (2016).
